# The relationship between self-confidence and attitude of emergency medical technicians towards family presence during resuscitation

**DOI:** 10.1186/s12245-024-00766-3

**Published:** 2024-12-03

**Authors:** Jaber Najafi, Neda Gilani, Hadi Hassankhani, Mansour Ghafourifard, Abbas Dadashzadeh, Mahnaz Zali

**Affiliations:** 1 Department of Medical Surgical Nursing, Faculty of Nursing and Midwifery, Tabriz University of Medical Sciences, Tabriz, Iran; 2 Department of Statistics and Epidemiology, Faculty of Health, Tabriz University of Medical Sciences, Tabriz, Iran; 3 Emergency and Trauma Care Research Center, Tabriz University of Medical Sciences, Tabriz, Iran; 4 Department of Medical Surgical Nursing, Faculty of Nursing, Qom University of Medical Sciences, Qom, Iran; 5 Medical Education Research Center, Health Management and Safety Promotion Research Institute, Tabriz University of Medical Sciences, Tabriz, Iran

**Keywords:** Cardiopulmonary resuscitation, Emergency Medical technicians, Family presence during resuscitation, Prehospital Emergency Care, Self-confidence

## Abstract

**Background:**

Family presence during resuscitation is a controversial issue worldwide. The aim of this study was to investigate the self-confidence and attitudes of Emergency Medical Technicians (EMTs) towards family presence during resuscitation (FPDR).

**Methods:**

In this cross-sectional study, a random sample of 252 EMTs were selected from 110 prehospital emergency centers. Two main questionnaires were used to collect data on the EMTs’ self-confidence and attitudes towards FPDR.

**Results:**

The results showed that the EMTs’ attitudes towards FPDR were lower than the mean (43.69 ± 19.40). In addition, more than 85% of them stated that the resuscitation process was stressful for the patient’s companions. There was a positive correlation between EMTs’ self-confidence and attitudes towards FPDR (*r* = 0.52, *p* < 0.01). The results showed that the smaller number of family members present during resuscitation was associated with higher EMTs’ self-confidence and more positive attitudes towards FPDR. Moreover, personnel with more experience, liability insurance, and advanced resuscitation training were significantly more self-confident than other personnel.

**Conclusion:**

A large number of the EMS personnel have a negative attitude towards FPDR, but EMTs, with higher self-confidence, have a more positive attitude. Therefore, it is possible to improve the EMTs attitudes towards FPDR and increase their self-confidence by training them to perform resuscitation in the presence of the family and by preventing people from gathering at resuscitation scenes.

## Introduction

Cardiac arrest is one of the main causes of death in thousands of people. Many of these cardiac arrests occur outside the hospital [1, 2]. Cardiopulmonary resuscitation (CPR) is the most important lifesaving technique which is performed in patients with cardiac arrest [3]. It involves a series of measures to restore the function of vital organs such as the heart and lungs, to prevent brain damage [4]. CPR is mainly performed by EMTs. These healthcare providers play a vital role in managing medical emergencies and saving the lives of patients around the world [5, 6].

The results of previous studies have shown that family presence during resuscitation (FPDR) is an integral part of Out-of-hospital cardiac arrest (OHCA) [7–9]. The results of a study of prehospital CPR in Iran showed that 83.1% and 11.5% of resuscitations were performed at the patient’s home and in public places, respectively [10]. FPDR is considered a highly controversial issue in the worldwide [11]. The EMTs face many challenges due to the need to perform CPR in the emergency situation Including; protecting themselves, dealing with unpredictable situations such as the accident or crime scene, having increased workload in a small team, dealing with the unreasonable expectations and requests of patients and family members [12–14], facing the risk of inappropriate reactions and disrespect [17], protecting the privacy of patients [15], performing futile resuscitation [16, 17]. These challenges may influence the performance and outcome of CPR [18].

Although there are many studies on the FPDR in the hospital setting, few studies have investigated on the FPDR in the prehospital setting. The result of a study in Turkey showed that 65.07% of Emergency Medical Services (EMS) personnel strongly disagreed with FPDR [9]. The results of a study in France showed that FPDR could affect the duration of CPR and the concentration of the EMS team [7]. Based on the results of a study conducted in the United States, many EMS personnel stated that they felt uncomfortable with FPDR [8]. A review of the relevant literature shows that few studies have been conducted on the attitudes and self-confidence of EMTs towards on the FPDR worldwide. On the other hand, there is no study that has investigated this phenomenon in EMS in Iran.

According to the literature review, healthcare provider’s self-confidence is one of the factors influencing the implementation of FPDR [19, 20]. The results of a study by Porter et al. showed that attending training courses and having self-confidence in emergency situations were necessary for the successful performance of FPDR [21]. In another study, Haririan et al. showed that nurses who had more negative attitudes towards FPDR had lower levels of self-confidence. On the other hand, the nurses who had more positive attitudes towards FPDR had higher levels of self-confidence and agreed with the presence of a family member during CPR [22]. In a study in Iran, Rafiei et al. reported similar findings [23]. In addition,, Twibell et al. [24] and Tudor et al. [25] found that nurses with higher levels of self-confidence had more positive attitudes towards FPDR.According to the literature review, there are many studies on the perception of FPDR by physicians, nurses and other health care providers in the hospital settings [26–29].

Studies conducted in different countries have shown that few studies have investigated EMTs perspectives on the FPDR [9]. In addition, EMTs’ self-confidence and attitudes towards FPDR have not been investigated in previous research. Considering the difference between hospital and pre-hospital settings, it is necessary to conduct this research. On the other hand, the different religious and cultural context in Iran and other Middle Eastern countries limits the possibility of using the results of similar studies conducted in countries with different contexts. Therefore, the purpose of this study is to investigate the relationship between self-confidence and EMTs’ attitudes towards FPDR.

## Methods

### Study Design and setting

This cross-sectional study was conducted on the EMTs of East Azarbaijan Province, Iran. East Azerbaijan Province is a province in northwestern Iran with an approximate area of 45 square kilometers. There are 76 urban and 54 intercity ambulance stations and one air emergency base in this province, with 586 pre-hospital personnel providing services. According to laws and policies based on Iranian culture in Iran, only men can work in the prehospital settings.

The EMS in Iran, a division of the Ministry of Health, provides free emergency services funded by the government. This system in Iran developed according to the American-British system but adapted some local conditions; it delivers care from the scene of injury, home, school, or other location and transports patients to medical centers. Emergency medical technicians and nurses work and collaborate with each other on missions. The dispatch center identifies emergencies and deploys the nearest team. Urban response times average 8 min, while road accident response averages 14 min. EMS staffs consult with physicians by phone when necessary, and the system operates independently from other emergency services, such as the Red Crescent, fire department, and police.

Participants in this study were selected using simple random sampling. Data collection took place May to September 2021. The inclusion criteria involved: giving informed consent to participation in the study and working in the prehospital emergency services with experience of performing CPR in the prehospital settings. participants were excluded if they did not have experience of performing CPR in the presence of a family member.

Using Krejcie and Morgan’s formula [30], 95% confidence, 5% error, and 20% ​​attrition rate, the sample size of the study was estimated to be 290. Due to the pandemic nature of COVID 19, an online based questionnaire was developed for data collection. specifically, the participants were provided with the link to the online questionnaire using social media applications or the email service. A reminder message was sent to the participants every 10 days in order to obtain the necessary samples.

### Measures

Three instruments were used for data collection. The first instrument was a demographic checklist, which examined the demographic and job characteristics of the personnel. The second instrument was a questionnaire that investigated the personnel attitudes towards FPDR. This questionnaire was first developed by Chi-chung [31]. It consisted of 22 items which examined the attitudes in 2 parts. The first part included 18 items on a 5-point Likert-scale (1 = strongly disagree to 5 = strongly agree). The lowest and highest scores on this questionnaire were 18 and 90, respectively.

The higher the score, the more positive the participants’ attitudes towards FPDR. In this questionnaire, 2 items focused on the patients’ rights regarding the presence of their companions during resuscitation. In addition, 7 items assessed the EMTs attitudes towards the benefits of FPDR. Finally, 9 items assessed EMTs attitudes towards the potential disadvantages and concerns of FPDR.

The second part of the questionnaire consisted of 4 items which were rated on a 5-point Likert scale (1 = not important, to 5 = very important). These items explored EMTs attitudes towards the importance of support requirements for implementing FPDR. The total score for this part of the questionnaire ranged from 4 to 20. The highest score indicated the most important pre-requisite for EMTs to implement the FPDR program.

The third questionnaire was the Family Presence Self-confidence Scale (FPS-CS). It assessed the self-confidence of the FPDR personnel. This 17-items questionnaire was developed by Twibell et al. (2008) [24]. The psychometric analysis of the Persian version of the questionnaire was carried out by Rafiei et al. [23] and Haririan et al. [22] in Iran. Responses were rated on a 5-point Likert scale (1 = strongly disagree to 5 = strongly agree). In this questionnaire, the lowest and highest scores were 17 and 85, respectively. The higher the score, the more confident EMTs were about the FPDR.

The content validity method was used to test the validity of the instruments. After translating the questionnaires using the forward-backward translation method, the questionnaires were sent to 10 nursing faculties and 15 experts in prehospital emergency care. The necessary corrections were then made based on the comments of the faculty members and experts. The reliability of the questionnaires was assessed using the method of internal consistency (Cronbach’s alpha). Based on the results, the reliability indices of the opinions on FPDR practice questionnaire and the self-confidence questionnaire were 0.85 and 0.92, respectively.

### Statistical analysis

The data collected were analyzed using IBM SPSS Statistics software (version 26). Descriptive statistics (i.e. frequency, percentage frequency, mean and standard deviation) were used to summarize the data. Pearson’s correlation coefficient and univariate and multivariate linear regression analyses was used to determine the relationship between the participants’ attitudes and self-confidence in FPDR. The significance level was considered to be less than 0.05.

## Results

Of the 290 questionnaires distributed, 252 were returned (response rate = 86.90%). The mean age of the participants was 35.54 ± 6.99 years, all were male, 64.7% had a bachelor’s degree, 92.9% had received advanced CPR training, and 94% (237 participants) had experience with FPDR. According to the results, 3 to 4 family members were present at most of the CPR scenes (Table 1).

**Table 1:** Attitudes and self-confidence of participants at the research sites based on demographic variables

Variable	Category	*N*	%	Attitude Mean (SD)	Self-confidence Mean (SD)
Age					
	< 30	54	21.4%	45.07(3.14)	66.67(2.79)
	30 to 40	121	48.0%	41.89(1.65)	66.53(1.41)
	> 40	77	30.6%	45.65(2.36)	64.53(1.71)
Marital Status					
	Single	67	26.6%	45.64(2.38)	67.75(2.04)
	Married	185	73.4%	43.06(1.48)	65.35(1.20)
Education					
	Diploma Degree	75	29.8%	42.38(2.46)	62.53(2.13)
	Bachelor’s Degree	163	64.7%	44.38(1.48)	67.55(1.17)
	Master’s and Ph.D. degrees.	14	5.6%	40.23(7.14)	63.62(5.45)
Discipline					
	Emergency Medical Technician	185	73.4%	44.51(1.43)	67.03(1.19)
	Nurse	48	19.0%	38.29(3.03)	62.84(2.58)
	Anesthesiology	14	5.6%	47.23(6.11)	64.84(4.17)
	Other	5	2.0%	55.51(6.78)	60.92(4.71)
Work experience in EMS					
	< 1 year	15	6.0%	45.12(7.70)	65.13(6.71)
	1–5 years	47	18.7%	44.04(3.60)	64.85(2.85)
	6–10 years	75	29.8%	42.21(1.95)	65.58(1.85)
	11–20 years	100	39.7%	44.49(1.92)	67.87(1.39)
	> 20 years	15	6.0%	43.88(5.28)	58.57(4.36)
Type of emergency department					
	City	178	70.6%	43.60(1.43)	66.95(1.26)
	Road	61	24.2%	43.02(3.07)	61.90(1.90)
	Village and Other	13	5.2%	48.24(4.76)	68.53(3.21)
ALS Training					
	Yes	234	92.9%	44.59(1.28)	67.20(1.04)
	No	18	7.1%	31.25(5.00)	48.56(2.90)
History of resuscitation in the presence of the patient’s family					
	Yes	237	94.0%	43.69(1.26)	65.94(1.03)
	No	15	6.0%	-	-
Number of times resuscitations with family present					
	< 10 person	70	29.5%	46.33(2.37)	63.48(1.65)
	10 to 20 person	59	24.9%	41.06(2.28)	64.72(1.68)
	21 and 30 person	27	11.4%	41.95(4.18)	67.41(3.84)
	31 and 40 person	24	10.1%	41.24(3.85)	70.26(3.29)
	> 40 person	57	24.1%	45.04(2.65)	67.72(2.45)
Number of patients’ family members present at resuscitation scenes					
	1 to 2 people	35	14.8%	49.04(3.15)	66.99(2.31)
	3 to 4 people	176	74.3%	44.82(1.44)	68.44(1.04)
	5 to 6 people	9	3.8%	23.81(5.30)	54.70(6.82)
	> 6 people	17	7.2%	31.57(3.60)	43.80(4.73)

The results showed that the standardized score of the EMTs attitudes towards FPDR was lower than the mean (43.69 ± 19.40). Therefore, EMTs had a relatively negative attitude towards FPDR. In this study, 97.5% of the EMTs believed that the CPR process was stressful for the patient’s family, and 86.9% of the EMTs stated that the patient’s family members intervened in their CPR process. (Table 2).

**Table 2:** Frequency distribution of selected items of EMTs’ attitudes towards FPDR (*N* = 237)

scale item Standardized attitude score Mean (43.69 ± 19.40).	Strongly disagree/Disagree (points 1–2), *N* (%)	Don’t know (Points 3), *N* (%)	Agree/strongly agree (Points 4–5), *N* (%)
CPR process too distressing for family members.	3 (1.3)	3 (1.3)	231 (97.4)
Family members may interfere on the CPR process	22 (9.3)	9 (3.8)	206 (86.9)
The presence of Family members will prolong CPR and make the decision to stop more difficult.	26 (11.0)	15 (6.2)	196 (82.8)
Emotional stress for EMTs’ is increased by the presence of Family members.	34 (14.3)	12 (5.1)	191 (80.6)
FPDR improves their understanding of CPR.	47 (19.8)	37 (15.6)	153 (64.6)
FPDR allows relatives to ensure that everything has been done.	59 (24.9)	22 (9.3)	156 (65.8)
FPDR makes them more ready to accept the patient’s death and eases the grieving process for the family.	56 (23.6)	31 (13.1)	150 (63.3)

The results showed that the participants’ standardized self-confidence score was higher than the mean (65.94 ± 15.89). In fact, the EMTs had a relatively high level of self-confidence in their ability to perform CPR in the presence of the patient’s family members. In this study, the majority of respondents indicated that they could easily perform CPR in the FPDR and 70% of them indicated that they could manage the CPR scene in the prehospital settings (Table 3).

**Table 3:** Frequency distribution of selected items of EMTs’ self-confidences towards FPDR (*N* = 237)

scale item Standardized self-confidence score Mean (65.94 ± 15.89)	Strongly disagree/Disagree (points 1–2), *N* (%)	Don’t know (Points 3), *N* (%)	Agree/strongly agree (Points 4–5), *N* (%)
I could perform chest compressions during CPR with FPDR.	7 (2.9)	8 (3.4)	222 (93.7)
I could give electrical therapies during CPR with FPDR.	14 (5.9)	19 (8.0)	204 (86.1)
I could administer drug therapies during CPR with FPDR.	8 (3.4)	26 (11.0)	203 (85.6)
I could identify family members who display appropriate coping behaviors to be present during resuscitation.	7 (3.0)	31 (13.1)	199 (83.9)
I could communicate about the resuscitation effort to family members who are present.	25 (10.6)	35 (14.8)	177 (74.6)
I could debrief the family after the resuscitation of their family member.	15 (6.3)	39 (16.5)	183 (77.2)
I could manage the CPR scene at the FPDR.	26 (11.0)	43 (18.1)	168 (70.9)

The results of the Pearson correlation coefficient indicated that there was a significant positive relationship between EMTs attitudes towards FPDR and their self-confidence in FPDR (*r* = 0.52, *p* < 0.01).

In this study, linear regression analysis showed that the smaller the number of family members present during the resuscitation, the more positive the EMTs’ attitudes towards FPDR. Those who encountered 5 to 6 family members at the scene of CPR had the most negative attitudes towards FPDR. Furthermore, EMTs working in small towns had more positive attitudes towards FPDR compared to EMTs working in large cities (*p* < 0.05) (Table 4).

**Table 4:** Relationship between EMTs’ individual- social characteristics and their attitudes towards FPDR

Variable	Category	Univariate Linear Regression		Multivariate Linear Regression	
		β (95% CI)	*P*	β (95% CI)	*P*
Work experience in EMS					
	< 1 year	1.25 (-15.20, 17.70)	0.881	-1.83 (-25.21, 21.55)	0.878
	1–5 years	0.16 (-11.62, 11.94)	0.979	-2.44 (-16.81, 11.93)	0.738
	6–10 years	-1.66 (-12.90, 9.57)	0.771	-3.71 (-16.65, 9.23)	0.572
	11–20 years	0.62 (-10.40, 11.63)	0.912	-6.48 (-17.63, 4.68)	0.254
	> 20 years	Reference		Reference	
Workplace (City)					
	Tabriz	-2.46 (-7.43, 2.50)	0.329	-7.17 (-13.28, -1.06)	0.022^*^
	Other Towns	Reference		Reference	
ALS Training					
	Yes	13.34 (3.58, 23.11)	0.008^*^	0.53 (-9.89, 10.95)	0.920
	No	Reference		Reference	
Liability Insurance					
	Yes	1.15 (-4.63, 6.94)	0.695	-1.33 (-7.00, 4.34)	0.644
	No	Reference		Reference	
Number of patients’ family members present at resuscitation scenes					
	1 to 2 people	17.46 (0.74, 26.43)	0.002^*^	0.66 (-10.27, 12.03)	0.909
	3 to 4 people	13.24 (1.56, 22.61)	0.006^*^	-3.21 (-13.77, 7.08)	0.539
	5 to 6 people	-7.76 (-22.96, 7.61)	0.315	-13.16 (-28.32, 2.00)	0.089
	> 6 people	Reference		Reference	
Self-Confidence		0.64 (0.51, 0.77)	< 0.001^*^	0.71 (0.54, 0.88)	< 0.001^*^

According to the linear regression analysis, the lower the number of family members present during CPR of patients, the higher the level of self-confidence of EMTs. The results showed that certain groups of EMTs, including those who had an advanced CPR certificate, liability insurance, a degree in emergency medicine, and 11 to 20 years of work experience, had significantly higher levels of self-confidence compared to other EMTs (*p* < 0.05) (Table 5).

**Table 5:** Relationship between EMTs’ individual-social characteristics and their self-confidence towards FPDR

Variable	Category	Univariate Linear Regression		Multivariate Linear Regression	
		β (95% CI(	*P*	β (95% CI(	*P*
Discipline					
	Emergency Medical Technician	6.11 (-8.08, 20.30)	0.398	13.62 (0.72, 26.53)	0.039^*^
	nurse	1.92 (-12.82, 16.66)	0.798	7.03 (-6.20, 24.30)	0.298
	Anesthesiology	3.58 (-12.72, 19.89)	0.665	9.62 (-4.98, 24.21)	0.195
	Other	Reference		Reference	
Work experience in EMS					
	< 1 year	6.56 (-6.80, 19.91)	0.334	7.85 (-11.20, 26.89)	0.418
	1–5 years	6.28 (-3.28, 15.85)	0.197	1.90 (-9.82, 63.13)	0.749
	6–10 years	7.01 (-2.11, 16.13)	0.131	3.10 (-7.45, 13.65)	0.563
	11–20 years	9.30 (0.36, 18.24)	0.042^*^	10.28 (1.28, 19.27)	0.025^*^
	> 20 years	Reference		Reference	
Workplace (City)					
	Tabriz	5.43 (1.41, 9.44)	0.008^*^	5.78 (0.86, 10.71)	0.022^*^
	Other Towns	Reference		Reference	
ALS Training					
	Yes	18.64 (10.88, 26.40)	< 0.001^*^	14.98 (6.73, 23.23)	< 0.001^*^
	No	Reference		Reference	
Liability Insurance					
	Yes	6.28 (1.61, 10.95)	0.009^*^	4.14 (-0.45, 8.73)	0.077
	No	Reference		Reference	
Number of patients’ family members present at resuscitation scenes					
	1 to 2 people	23.19 (14.74, 31.63)	< 0.001^*^	21.49 (12.70, 30.28)	< 0.001^*^
	3 to 4 people	24.64 (17.39, 31.90)	< 0.001^*^	24.79 (17.12, 32.46)	< 0.001^*^
	5 to 6 people	10.90 (-0.87, 22.67)	0.069	11.22 (-1.06, 23.50)	0.073
	> 6 people	Reference		Reference	

In addition, the results of univariate linear regression indicated that there was a significant relationship between EMTs’ attitudes towards FPDR and their self-confidence. Specifically, on average, a one-unit increase in self-confidence score associated with a 0.64-unit increase in attitude score towards FPDR (*p* < 0.001).

## Discussion

This is the first study to investigate the relationship between EMTs’ self-confidence and attitudes towards FPDR in the prehospital settings. Findings are consistent with previous studies showing that EMTs have a high level of experience of FPDR [7, 8, 32]. Therefore, these experiences can be used to develop training guidelines for personnel to perform safe CPR and support patients’ families in the prehospital settings.

The results showed that personnel attitudes towards FPDR were lower than the mean. These results are in line with the results of studies conducted in Turkey [9] and Portugal [32]. Furthermore, the results of other studies have indicated that most of the EMS teams do not prefer to perform CPR in the presence of the patient’s family members and a large number of them feel uncomfortable [7, 8]. This attitude may affect the physical and mental health and performance of the EMTs, as well as the quality of CPR outcomes [7–9]. Therefore, it is necessary to include training in the performance of CPR in the presence of the patient’s family, the management of resuscitation scenes, in the national standard curriculum for EMTs.

In this study, family stress and interference, prolongation of CPR time, the difficulty in decision making to stop CPR, and emotional stress of the Personnel were the most important factors causing the negative attitude of the EMTs towards FPDR. These results support the findings of the studies conducted by Amaral et al. [32] and Belpomme et al. [7]. Likewise, some other studies have reported that the personnel’s feeling of being under surveillance [9, 33], the family members’ interference in the personnel’s work [8, 32] and the family members’ stress [32] are the factors that cause the personnel to have a negative attitude towards FPDR. Of course, there is the same concern and fear of being filmed by the family or other people in our country (Iran). Therefore, the EMS staff try to provide high-quality care and work towards gaining the trust of patients and their families. Moreover, they quickly transfer the patient to the ambulance and provide specialized care within the ambulance, thus alleviating these concerns. In order to deal with these issues, it is possible to take certain measures. These measures include informing the patient’s family members about the conditions of CPR, using cubicle curtains, and getting help from the experienced people and health care providers at the scene. These measures can reduce the family stress, improve the situation and prompt personnel to adopt a more positive attitude towards FPDR.

This study was the first to investigate EMTs’ self-confidence in their ability to perform CPR in the FPDR. The results of the study showed that the self-confidence of the personnel was higher than the mean (65.94 ± 15.89). In this regard, in a study of in-hospital nurses in the state of Kentucky, the self-confidence score was 61.2 ± 1.19 [25]. Moreover, the above-mentioned score was 53.86 ± 11.7 [23] and 52.91 ± 12.69 [22] in the studies of nurses in the special care units in Qazvin (Iran) and nurses in teaching hospitals in Tabriz (Iran), respectively. These results are in line with the findings of the present study. Having high self-confidence in pre-hospital CPR is the strength of the EMTs, which is necessary to manage the CPR scene. Therefore, it is recommended to identify the factors involved in increasing the self-confidence of the EMTs.

In this study, the EMTs’ highest self-confidence score was related to their ability to perform cardiac compressions and to give electric shocks to patients. This result shows that the EMTs implemented the AHA recommendations [34]. Furthermore, EMTs concentrate all their efforts on the patients’ resuscitation and do not pay attention to their family members [35]. Therefore, it is recommended that EMTs receive training in communication skills. The results showed that there was a significant correlation between the personnel attitudes towards FPDR and their self-confidence in their ability to perform CPR in the presence of the patient’s family. The higher the EMTs self-confidence score, the more positive their attitude towards FPDR. These findings support the results of a study conducted by Twibell et al. which showed that nurses with higher levels of self-confidence had more positive attitudes towards FPDR [24]. Likewise, the results of a studies conducted in Australia [36], the United States of America [25], Jordan [37] and Iran [22, 23] showed that there was a relationship between the hospital nurses’ attitudes towards FPDR and their self-confidence. Therefore, it would seem that training EMTs to have a positive attitude towards FPDR will lead to an increase in their self-confidence in performing CPR.

The results of the present study indicated that the EMTs who had received advanced CPR training had higher levels of self-confidence and more positive attitudes towards FPDR. In this regard, the results of the previous studies have shown that the personnel training leads to an increase in their self-confidence to perform resuscitation and improves their attitudes towards FPDR [25, 38]. In addition, the results showed that, the more experienced personnel had higher levels of self-confidence. This finding supports the results of previous studies [38–40]. Therefore, continuous training of EMTs based on the latest CPR guidelines and the use of experienced personnel for out-of-hospital resuscitation is recommended.

One of the unique results of the study was that the lower the number of family members’ presence during resuscitation, the more positive the EMTs’ attitudes towards FPDR and the higher their self-confidence. In this context, it is true that is recommended to facilitate the presence of the family with the patient [20], but it is necessary to prevent the gathering of many people in order to manage critical scenes as well as possible [18]. It seems that it is a necessary to take a number of measures to perform the resuscitation of patients away from crowded places. These measure may include informing people about the CPR process to prevent them from gathering at the scenes, transferring patients to the ambulance, using cubicle curtains, getting help from experienced individuals, and asking the patient’s other family members to leave the CPR scenes.

### Limitations

This study had several limitations. It was conducted in one province in the northwest of Iran, which may affect the generalizability of the findings. Therefore, the results should be interpreted with caution as they may not apply to all prehospital personnel in Iran or to countries where EMTs provide prehospital care.

## Conclusion

A large number of the EMS personnel have negative attitudes towards FPDR. The main reasons for these negative attitudes are the increase in stress for the personnel and patient’s family, the interference of family member in the resuscitation process, and the prolongation of the CPR operations. Considering the constant presence of the patient’s family members in the prehospital resuscitation process, it is a necessary to take certain measures to manage the FPDR in an effective way and to facilitate the care of the patients in the presence of their family members.

In this regard, it is possible to reduce the stress of the patient’s family members by identifying their expectations and observing their behavior, supporting the patient’s family members during their CPR, and providing the patient’s family members with a suitable environment. Moreover, it is possible to improve the EMTs’ attitudes towards FPDR and increase their self-confidence in their ability to perform CPR on patients in FPDR by developing training guidelines for the EMTs, recruiting EMTs experienced in CPR conditions, ensuring the timely presence of support teams, getting help from experienced individuals, and preventing people from gathering at CPR scenes. Based on the results, it can be argued that FPDR and the performance of CPR by members of the emergency medical services are important issues that need more attention in prehospital resuscitation.

## Data Availability

The datasets analysed during the current study are available from the corresponding author on reasonable request.

## Abbreviations

FPDR: Family presence during resuscitation
EMS: Emergency medical services
EMT: Emergency medical technician
CPR: Cardiopulmonary resuscitation
